# Good death: An exploratory study on perceptions and attitudes of patients, relatives, and healthcare providers, in northern Tanzania

**DOI:** 10.1371/journal.pone.0233494

**Published:** 2020-07-10

**Authors:** Temitope O. Gafaar, Msafiri Pesambili, Oliver Henke, Joao Ricardo Nickenig Vissoci, Blandina Theophil Mmbaga, Catherine Staton

**Affiliations:** 1 Duke University School of Medicine, Duke University, Durham, NC, United States of America; 2 Duke University Research Collaboration, Kilimanjaro Christian Medical Center, Moshi, Tanzania; 3 Cancer Care Center, Kilimanjaro Christian Medical Center, Moshi, Tanzania; 4 Duke Global Health Institute, Duke University, Durham, NC, United States of America; 5 Duke Emergency Medicine, Duke University Medical Center, Durham, NC, United States of America; 6 Kilimanjaro Clinical Research Institute, Moshi, Tanzania; ESIC Medical College & PGIMSR, INDIA

## Abstract

**Importance:**

In the Kilimanjaro region of Tanzania, there are no advance care planning (ACP) protocols being used to document patient preferences for end-of-life (EoL) care. There is a general avoidance of the topic and contemplating ACP in healthcare-limited regions can be an ethically complex subject. Nonetheless, evidence from similar settings indicate that an appropriate quality of life is valued, even as one is dying. What differs amongst cultures is the definition of a ‘good death’.

**Objective:**

Evaluate perceptions of quality of death and advance EoL preparation in Moshi, Tanzania.

**Design:**

13 focus group discussions (FGDs) were conducted in Swahili using a semi-structured guide. These discussions were audio-recorded, transcribed, translated, and coded using an inductive approach.

**Setting:**

Kilimanjaro Christian Medical Centre (KCMC), referral hospital for northern Tanzania.

**Participants:**

A total of 122 participants, including patients with life-threatening illnesses (34), their relatives/friends (29), healthcare professionals (29; HCPs; doctors and nurses), and allied HCPs (30; community health workers, religious leaders, and social workers) from KCMC, or nearby within Moshi, participated in this study.

**Findings:**

In characterizing *Good Death*, 7 first-order themes emerged, and, of these themes, *Religious & Spiritual Wellness*, *Family & Interpersonal Wellness*, *Grief Coping & Emotional Wellness*, and *Optimal Timing* comprised the second-order theme, *EoL Preparation and Life Completion*. The other first-order themes for *Good Death* were *Minimal Suffering & Burden*, *Quality of Care by Formal Caregivers*, and *Quality of Care by Informal Caregivers*.

**Interpretation:**

The results of this study provide a robust thematic description of *Good Death* in northern Tanzania and they lay the groundwork for future clinical and research endeavors to improve the quality of EoL care at KCMC.

## Introduction

Due to advancements in modern medicine, especially intensive care treatments, the process of dying is happening over longer periods of time and an increasing number of people require long-term care for chronic conditions [1, 2]. Furthermore, the world’s population is aging and there has been a steady rise in annual deaths worldwide [3]. In high-income countries (HICs), advance care planning (ACP) has been increasingly promoted since the 1990s [4] due to mounting awareness about the poor quality of end-of-life (EoL) care and the poor knowledge of patients’ preferences for EoL treatment decision-making [5, 6]. ACP is a dynamic *process* whereby individuals are able to explore, clarify, and describe their goals and preferences for future medical care [7].

Approximately 80% of annual deaths worldwide occur in low-and-middle countries (LMICs) [8]. However, the most developed ACP provision services occur in HICs and most of the published data on ACP originate from such resource-rich regions [7]. There have been scarce studies on planning for EoL care in sub-Saharan Africa (SSA) [9]. At Kilimanjaro Christian Medical Centre (KCMC), the referral hospital for northwestern Tanzania, there is no tool or protocol to assess an individual’s wishes and priorities for EoL care. Due to cultural perceptions of death as a taboo subject in northern Tanzania, there is a general avoidance of advance care planning conversations [10]. Similarly, in other comparable settings, planning for EoL care is not common due to strong prohibitions against talking about death, which is considered highly distressing and/or associated with the fear that death talk will accelerate death [11–13]. Furthermore, the ethical framework for modern advance directives was derived from Western principles, which place a high value on individual autonomy [14, 15]. In contrast, in many non-Western cultures, whereby a dying person is largely regarded within the context of strong relational ties, family and community are considered the source of treatment decisions, more so than the autonomous individual [16, 17].

Despite these concerns, there is substantial evidence from resource-limited settings that people value an appropriate quality of life, even as they are dying [18–22]. And there is evidence that indicates interest in planning for EoL care in non-Western nations [23]. When considering the appropriateness of introducing an ACP protocol at KCMC, it was evident that there was a literature deficit on the cultural views of quality of death (QoD) in Tanzania but two previous studies from northern Tanzania had indicated that QoD was an existing concept in northern Tanzania. In the 2002 report for Harris et al.’s qualitative study on physician disclosure of cancer diagnosis, there’s a brief mention of a good death [17] and a 2008 ethnographic study from the Mara region of northwest Tanzania, described the impact of HIV/AIDS stigma on QoD [21]. However, there had been no study to carry out a robust QoD descriptive analysis and it was clear that addressing this knowledge gap would be crucial for understanding the acceptability of ACP in this setting.

This study aims to evaluate perceptions of QoD and EoL advance planning amongst patients with life-threatening illnesses, their family or friends, healthcare professionals (HCPs; nurses and doctors), and allied healthcare professionals (allied HCPs; social workers, community health workers, and religious leaders), in northern Tanzania.

## Methodology

### Setting and ethics approval

KCMC, located in Moshi (population: 184,292 [24]), is the zonal referral hospital for northwestern Tanzania and the third largest hospital in the country [24]. The study was approved by the Institutional Review Board of Duke University, the Ethics Committee of KCMC and the National Institute for Medical Research, Tanzania. Written and/or verbal consent was obtained from all participants in Swahili (Appendix 1 in S1 File).

### Study design

The data was collected over a six-week period from January–March 2018 and reported based on the Consolidated Criteria for Reporting Qualitative Research [25]. Due to the exploratory nature of this study, we used the Grounded Theory qualitative approach, which we chose because prior literature was insufficient to develop a cultural framework for death and EoL care planning in northern Tanzania. The Grounded Theory method enables us to achieve conceptual understanding of death and ACP based on themes derived from (or ‘grounded’ in) the collected data [26, 27].

### Focus group guide

Discussions were facilitated using focus groups (FG). The focus group prompts were based on expert input and literature review during the study preparation phase. Expert panel included KCMC’s Palliative Care service, local healthcare providers, and researchers with qualitative research experience. Vignettes were used to help normalize the topic, some of which were adapted from the FG scripts used in Lewis et al.’s study, with author permission [10]. Other noteworthy factors and concepts that influenced the creation of the FG guide include: 1) predominant cultural values that prioritize community over self [28–31]; 2) the significant role of family in a patient's EoL care [10, 29]; and 3) physician reluctance to “break bad news” [10, 17]. See Appendices 2–4 in S1 File for the full FG guides.

After a re-iterative translation and back translation protocol, a set of focus groups were conducted with bilingual researchers and/or research nurses to analyze the theoretical and content evaluation of the translated FG guide. This served to verify: (a) the practical relevance, (b) language clarity, and (c) theoretical coherence of the items. Opinions were discussed collectively in the focus group sessions to address discordance and improve the quality of the translations.

### Study population

Focus groups were separated by participant type: patients, relatives, medical professionals, and allied HCPs. With the exception of some of the allied health professionals who had to be recruited from other locations within Moshi, all study participants were recruited by our research nurses from the various departments and clinics at KCMC.

#### Patients and family/friends

It was crucial that we recruited patients who had been confronted with EoL and who would be able to provide vital information on what northern Tanzanians value/prioritize near the end of life.

Participants were ≥18 years of age and had, or were related/close to somebody who had a life-limiting illness, specifically: a life-threatening injury, HIV/AIDS, malignancy, or advanced organ failure [32]. They had to be aware of their prognosis and be willing and able to talk about their experiences. In addition to being medically stable and able to communicate in Swahili and/or English, they also had to have decision-making capacity and be able to provide consent.

#### Healthcare professionals

Participants were ≥18 years of age. Due to their limited availability, HCPs were split into medical professionals (nurses and physicians) and allied HCPs (social workers, religious leaders, and community health workers).

### Procedure and data analysis

FGDs were conducted in Swahili and led by trained research nurses. All discussions were audiotaped, transcribed, and translated by bilingual Tanzanian natives with research experience.

Analysis: The English-translated FGD transcripts were coded for text related to *Good Death*, using NVivo 11.4. 13 focus groups (122 participants) were conducted and data saturation (i.e. no new codes emerged, and no new themes identified) was achieved by the 11^th^ focus group. Each transcript was coded inductively by two research authors, TG and MP–a bilingual Tanzanian native. The codes were compared, and discrepancies were resolved through discussion between the initial coders. First-order themes were identified by grouping similar codes and, similarly, second-order themes were derived from first-order themes. Responses from the different participant groups were also examined to identify any similarities, differences, and potential insight.

After each transcript was openly coded, a broad aggregation scheme was constructed iteratively based on emerging themes [27]. The final descriptive framework incorporates the aggregation scheme, the coding memos, and the focus group debrief notes. Select references that portray the meaning of each theme are included in this report.

## Results

Participants were patients with life-threatening illnesses (6–10 per group), their relatives/friends (9–10 per group), HCPs (14 doctors and 15 nurses; 8–11 per group), and allied HCPs (13 community health workers, 9 religious leaders, and 8 social workers; 10 per group). The mean age was 43.1 years and 63% (77) of the respondents were female. See Table 1 for more details.

**Table 1:** Baseline characteristics.

VARIABLES	Patients	Family or Friends	Medical Professionals	Allied Healthcare Professionals	All Participants
**No. of Participants, N** (% of total number of participants)	**34** (28)	**29** (24)	**29** (24)	**30** (24)	**122**
**Range of Participants per Focus Group**	**6–10**	**9–10**	**8–11**	**10**	**6–11**
**No. of Focus Group Discussions**	**4**	**3**	**3**	**3**	**13**
**Female, N** (%)	**24** (71)	**20** (69)	**17** (59)	**16** (53)	**77** (63)
**Age, Mean** (SD)	**49.4** (12.3)	**44.4** (13.1)	**36.4** (11.9)	**41.2** (16.8)	**43.1** (14.3)
**Age Ranges, N** (% of no. of participants in each group)					
< 21	**0**	**0**	**0**	**1** (3)	**1** (1)
21–35	**4** (1)	**8** (28)	**18** (62)	**12** (40)	**42** (34)
36–50	**14** (41)	**13** (45)	**5** (17)	**6** (20)	**38** (31)
51–65	**14** (41)	**7** (24)	**6** (21)	**9** (30)	**36** (30)
> 65	**2** (6)	**1** (3)	**0**	**2** (7)	**5** (4)

## Grounded theory analysis

Many participants held the sentiment that death is generally unwanted but certain; and they endorsed a belief that QoD can be differentiated into good or bad.

*"Good death… is when a person is prepared physically, spiritually, and mentally. For example, the patient has received treatments. They were close to their Imam (if they were Muslim) or to their pastor (if they were Christian). Psychologically, maybe they were cared for by their family and got help to leave their things in order; maybe they left a will and there was no conflict amongst their relatives or their children after they died. That is when we say someone has died a good death…they left their family in proper order, with future directions and without conflicts.*”

–*Allied Health Professional*. *Insert 1*

### Good death themes

**Fig 1:**
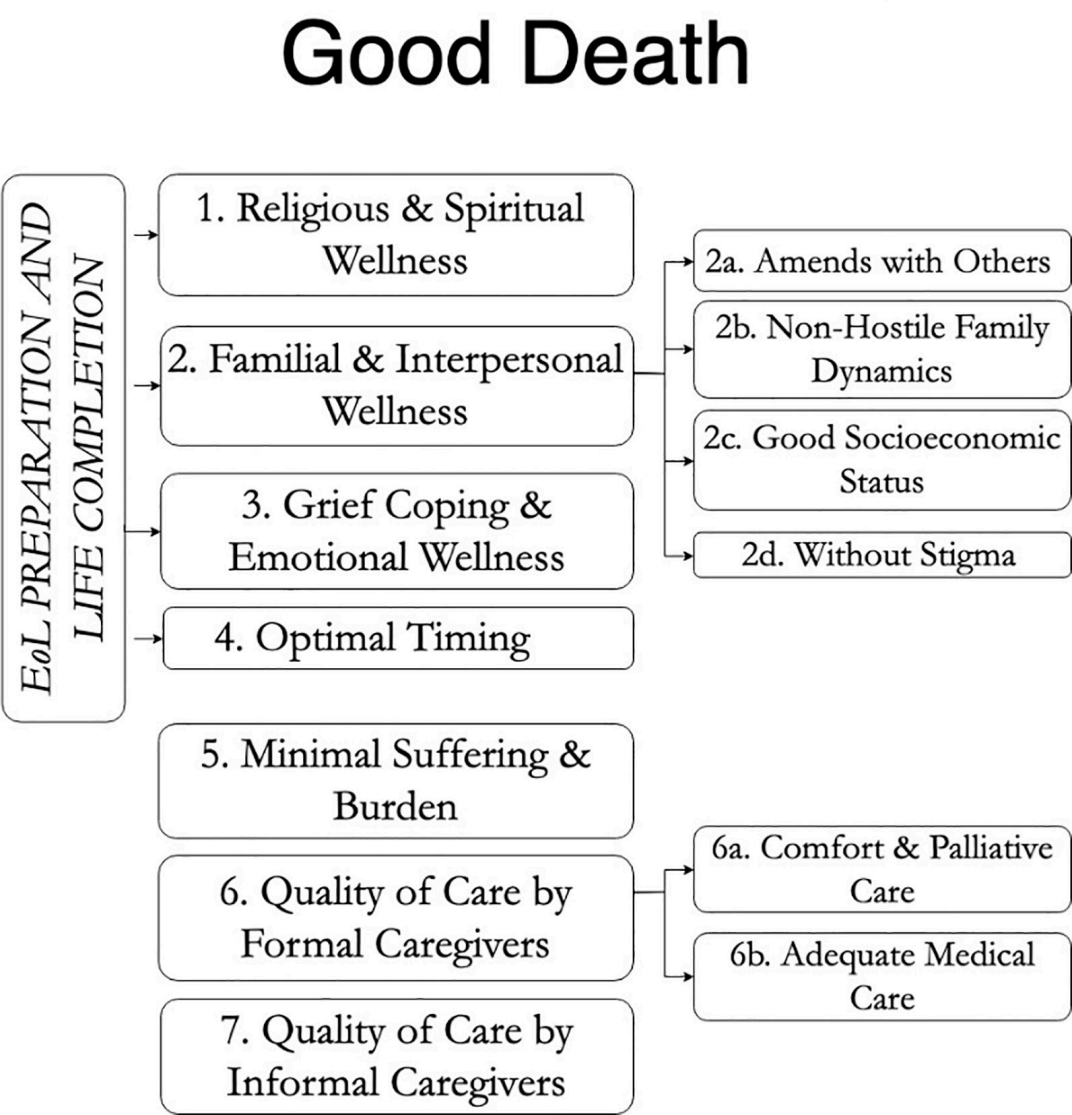
Grounded theory framework for good death. From left to right: second-order themes, first-order themes, and sub-themes.

For the characterization of *Good Death*, 7 first-order themes emerged. Of these, 4 –*Religious & Spiritual Wellness*, *Familial & Interpersonal Wellness*, *Grief Coping & Emotional Wellness*, and *Optimal Timing—*comprise the religious, existential, and psychosocial needs that were perceived to be important for Good Death and they make up the second-order theme, ‘EoL Preparation and Life Completion’ (Fig 1). Their impact on QoD can be interpreted as direct responses to the conditional statements:

- How well did one live with God and with others? *and*
- How well did one leave his/her affairs, family, and community behind?

*"in my opinion, good or bad death does not end with the cause of death*. *An example of a good death is when one leaves his community in a good condition …”*

*–Allied Health Professional*. *Insert 2*

"the worst death is that [you] do not prepare yourself.”

*–Family member*. *Insert 3*

All the themes associated with *Good Death* reflect a shared common ground and all were endorsed by patients, family/friends, HCPs, and allied HCPs. However, general distinctions in participants’ focus emerged and these appear to be influenced by socio-professional roles.

### EoL preparation and life completion

#### 1. Religious & spiritual wellness

There was a consensus amongst participants that maintaining a good personal relationship with God is important for *Good Death*. Participants also emphasized the importance of religious leaders and faith-based communities as these groups provide support to a dying person and family by offering prayers, visiting frequently, providing counsel, alleviating fears, and more.

Interviewer: What is a priority for the remainder of his life?

Participant: “It is to be spiritually and mentally prepared and, also, we will make arrangements for him to leave peacefully like calling a pastor or a sheikh, to pray for him.”

*–Medical Professional*. *Insert 4*

Amongst the participant groups, patients and religious leaders were more likely to focus on issues of religion and spirituality. There was a notable reference about the personal conflict that religious leaders face when trying to comfort patients while also avoiding the bias to view death as the enemy.

*"The first spiritual responsibility is to bolster the faith of the dying person and his family, but it is also important to be honest*. *Now, I will say that we, pastors, often give people false hope by lying. For example, you think somebody is near the end of life but you are telling him that Jesus will make him recover…. I want to say that we should be truthful. We are not God but when we see somebody that seems to be dying, we should be honest. Let us…acknowledge that death is inevitable but, also, continue to comfort the family and [encourage them to] avoid seeing death as an enemy.”*

*–Allied Health Professional*. *Insert 5*

#### 2. Familial & interpersonal wellness

From the discussions, it was evident that positive family and community relationships are critical for a good QoD. Participants expressed sentiments that seeking forgiveness, forgiving others, unburdening secrets, and maintaining good relationships with relatives are important actions for *Good Death*.

“…If a person has a good relationship with their community and their family and their church, we say he has died a good death.”

*–Patient*. *Insert 6*

Avoidance of family disputes was endorsed as a vital component of *Good Death* and participants often promoted the importance of keeping one’s family in order and peace. In particular, doctors and nurses were the only group to bring up EoL decision-making conflict within the context of caring for a patient on life-support. In hypothetical scenarios where decisional conflicts arose after an individual lost decision-making capacity or had passed away, the QoD was negatively perceived.

*"But a bad death is when a patient is already in a critical stage and isn’t prepared. He continues with treatment even though the relatives know that this person’s life is coming to an end, but they do not prepare him*. *Others continue to insist that he should continue with treatment. And, at the end of the day the patient dies painfully because his close relatives did not disclose his prognosis to him.”*

*–Medical Professional*. *Insert 7*

#### 3. Grief coping and emotional wellness

Mental preparedness and sound emotional well-being were endorsed as characteristics of *Good Death*; and ‘accepting death’, ‘having no fear’, ‘being with hope’, and ‘having peace of mind’ were some of the phrases that were associated with a good QoD. Responses also indicated a general belief in the concept of life after death and it was associated with *Good Death* due to its effects in alleviating fears and concerns about the finality of death.

*“Good Death is when … you get the opportunity to prepare your soul spiritually, like confessing your sins and repenting*, *and you are ready for the “new life” as we believe.”*

*–Medical Professional*. *Insert 8*

#### 4. Optimal timing

The duration of illness and the dying process played a crucial role in the perception of QoD, and this was because of its impact on a person’s ability or opportunity to be prepared for end-of-life. *Good Death* was often viewed as being dependent on having enough time to prepare one’s self and one’s family.

*"a good example is somebody who was sick for a long time and was able to be treated at a hospital*. *He had time to be cared for by his relatives and he had time to offer words of wisdom to his family and to get his affairs in order. “*

*–Allied Health Professional*. *Insert 9*

However, in characterizing *Bad Death*, participants’ responses revealed that the impact of the duration of illness and dying process could be viewed in two ways: it could be too short, and, thus, not allow an individual to be ready, or it could be prolonged–consequently increasing the illness burden for patient and family.

### Other good death themes

#### 5. Minimal suffering and burden

In describing QoD, the study subjects underscored the importance of considering characteristics of the illness journey, the environment of dying, and the ramifications on patient, family, and community. A minimal disease burden, e.g. being pain-free or dying during sleep, was associated with *Good Death*. Participants also associated not feeling like a burden on one’s relatives or informal caregivers with a good QoD. Conversely, significant or prolonged pain/disability (physical or non-physical) were associated with *Bad Death*.

*“…good death is when a person does not suffer for a long time*. *If it is an illness, they suffer little pain.”*

*–Patient*. *Insert 10*

Doctors, nurses, and community health workers were more attuned to matters of physical suffering and comfort care, especially pain relief.

#### 6. Quality of care by formal caregivers

Participants agreed that death was inevitable, but when elaborating on a good QoD, it was often described as one that occurs after appropriate and adequate medical efforts have been attempted. Adequate medical care encompasses the availability of good-quality medical care, a patient’s’ ability to access such care (i.e. not limited by location or finances), optimal utilization of modern medicine to the best of its abilities, and positive experiences with HCPs.

On the other hand, detrimental experiences with modern medicine—such as inadequate palliative and non-palliative medical care, limited resources, and adverse HCP encounters—contributed to the perceptions of *Bad Death*. A noteworthy response highlights a personal conflict that medical professionals may face when conducting aggressive therapies.

*“With regard to pain, we as health workers, sometimes contribute to a bad death. For example, we know that the possibility of recovery [from a certain ailment] is zero, but we find ourselves intubating patients, performing CPR, and we know the patient will not recover*. *We go to these lengths to appease the relatives. Generally speaking, if you know the patient will not recover it is better to leave them to die slowly and calm. Good death is that you die without pain”*

*–Medical Professional*. *Insert 11*

#### 7. Quality of care by informal caregivers

This theme elaborates on the impact of relatives and community members within their roles as informal caregivers. A positive QoD was associated with being cared for and comforted by loved ones, especially near EoL. Conversely, neglect, abuse, lack of support, and poor care provision, by one's family and/or community, were associated with a worse QoD.

*"A bad death, from my experience in patient care… is when a patient lacks help from their family, from the society and they fail even to get help from the hospital*. *They get to the point of, maybe, being bed-ridden and paralyzed and unable to fend for themselves. They do not have food; people have neglected them…"*

*- Allied Health Professional*. *Insert 12*

## Discussion

To the best of our knowledge, this is the first study to carry out an in-depth exploratory analysis on the conceptualization of *Good Death* in Tanzania. Our study revealed that, in northern Tanzania, the degree of readiness and closure has significant impact on how death is perceived. Death and suffering are highly individualized processes and *Good Death*, as elaborated upon in this paper, is not a morally absolute or monolithic concept, nor is it meant to be restrictive. Rather, our results provide a contextual framework for understanding what patients and their loved ones prioritize at the end of life. Clinically, these results may serve as a starting point for the dynamic process of assessing and addressing the physical, psychosocial, religious, and existential needs of dying patients and their families. Globally, our study methods can be employed in other resource-limited settings to characterize cultural perceptions of QoD and the priorities for EoL care.

Most of the descriptive analyses on QoD have been from HICs and, despite differences in historical, political, cultural, and epidemiological contexts [5], there are considerable similarities between previously-reported QoD descriptive models and our model from Tanzania. A 2008 review of published studies on QoD (n = 17) reported seven broad domains that were consistently identified by patients, families, and HCPs [33], all of which are featured in our QoD model. In a later review of 36 studies that evaluated good death perceptions, Meier et al. identified 11 core themes, including preferences for a specific dying process (including preferences for place of death), sound emotional well-being, pain-free status, and, notably, treatment preferences [34]. A 2017 meta-synthesis of 14 qualitative studies that evaluated good death from the bereaved relative’s perspective, most from HICs, showed that components of a good death experience included, amongst other factors, preparation for death, pain and symptom management, and place of EoL care [35]. However, in our study, specifying, or being able to specify, one’s preferences for treatment options and/or place of death was rarely brought up in the characterization of *Good Death*. This contrast possibly reflects the unattainability of aggressive therapies or inpatient hospitalizations in resource-limited settings like ours [34].

Portrayals of religion, religious leaders, and faith-based communities indicate that they are crucial components of one’s illness journey, dying process, and death aftermath (e.g. funerals and family bereavement). Our findings parallel numerous reports from other nations that have highlighted the importance of positive religious well-being at the end of life [13, 36–39]. A 2007 systematic review on the influence of religion on bereavement reported that 94% of the analyzed studies showed a positive effect [40]. According to a 2013 Botswana study, informal caregivers for dying loved ones who were visited by a religious leader near the end of life were more likely to rate the death experience positively [39]. Specifically, in northern Tanzania, the importance of religious coping during one’s illness journey seemingly cannot be overstated. A 2014 study on HIV/AIDS stigma concluded that religious coping was the predominant variable that was associated with acceptance of one’s HIV status [41]. Similarly, respondents in our study endorsed the perspective that religion provides comfort to patients, and their families, and helps them cope.

One of the ways religion impacts QoD and coping is through a shared belief in an afterlife. ‘Being with hope’ was associated with a good QoD and, in addition to capturing a sense of optimism for “the patient to live as well as possible until they die” [42], it also conveys a positive expectation for the afterlife. This is similar to what was reported in a study of good death in rural Kenya, “patients talked of ‘going home’ to a place without pain and suffering, or talked of going to heaven…” [13]. This notion is also supported by the results from a study on palliative care awareness amongst religious leaders in Kenya–participants highlighted how they were able to provide hope to patients by leveraging a belief in an afterlife [43]. Ultimately, it is important to bear in mind the complexity of the relationship between religion and bereavement [40, 44], but our findings indicate a positive association between belief in an afterlife and coping/bereavement in northern Tanzania.

Similar to multiple studies out of sub-Saharan Africa, participants’ responses emphasized cultural values that prioritize community and family above self [28–31]. Our data suggests that the responsibilities and burdens of informal caregivers influence how QoD is perceived and respondents detailed the multi-dimensionality of caregiver burden–financial, physical, social, and psychological. The financial component has been captured in previous studies and it encompasses the cost of providing the patient’s basic necessities and healthcare-associated needs [16, 17, 45–47], funeral costs [46], and the opportunity costs of time spent on care provision [45, 47]. Also similar to prior reports, when discussing EoL care, participants in our study portrayed the duality of the informal caregiver role–i.e. providing care and needing care [48]. They also highlighted the importance of supporting informal carers in various ways such as offering respite care and providing education on patients’ home medical management.

### ‘Being prepared’ for end of life

With this study, we set out to explore perceptions of *Good Death* and the role of EoL preparedness in northern Tanzania. Several characteristics of *Good Death–*such as having maintained a positive relationship with God, having made amends with family, or having provided clarity on economic matters to prevent conflict–involved planning in advance and/or having the opportunity to plan. The results indicate an overall positive perception of EoL advance planning; these sentiments have been echoed in multiple studies from HICs and they have contributed to the increasing awareness of ACP in those settings [5, 34, 49–53]. Furthermore, data from HICs argue that ACP potentially leads to: increased quality of life for patients and families [54]; higher satisfaction with care quality [4]; better family preparation on what to expect [4]; lower risk of stress, anxiety, and depression in surviving relatives [55]; and reduced EoL hospitalizations and intensive treatments [56, 57]. Importantly, emerging data indicate that ACP decreases cost of EoL care [57, 58], *without increasing mortality* [58].

However, the issue of introducing ACP in healthcare-and-resource-limited environments is ethically complex [5, 11]. Poverty is a common barrier to treatment [59–61] and settings like ours are limited in the ability to offer cutting-edge or aggressive life-saving interventions. Given the apparent need for improvement in intensive care and other aspects of healthcare, the relevance of understanding priorities for EoL care may seem obscure. However, we propose that these objectives are not mutually exclusive. Studies from multiple LMICs have shown that people desire a good quality of death and there is literature precedent for interest in the concept of ACP from LMICs [11, 23, 62]. Similarly, participants’ responses in our study conveyed a positive association between *Good Death* and *EoL Preparation and Life Completion*.

### Limitations

The focus group guides acknowledge the uncertainty of death and the predominant belief in God amongst Tanzanians [63–65]; this was because information gathered from the research nurses and other Tanzanian natives strongly suggested that approaching the topic of death from this perspective would encourage discussion. This is an important limitation of the study, but we felt that this was a culturally appropriate approach to address the issue considering that the topic is taboo in Tanzania [10]. Even though there was significant variability in our participant sample, especially with the illness diagnoses and the inclusion of allied health professionals, we acknowledge that not involving lay community members limits our understanding of QoD in Moshi. In addition, traditional medicine was rarely brought up during the focus group discussions despite > 50% of people in northern Tanzania accessing this type of care [66]. Participants’ reluctance to discuss it likely stems from social desirability bias, especially as the discussions were conducted on a hospital campus. We recommend further studies to evaluate the relationship between traditional medicine and QoD.

## Conclusion

Prior to this study, knowledge about QoD and preparedness for EoL in northern Tanzania was significantly lacking. From the grounded theory analysis of FGDs, we identified 7 first-order themes that characterized the perception of *Good Death*. Of these themes, *Religious & Spiritual Wellness*, *Family & Interpersonal Wellness*, *Grief Coping & Emotional Wellness*, and *Optimal Timing* comprised the second-order theme, *EoL Preparation and Life Completion*. The data indicates a positive perspective on preparing in advance for EoL care and appropriately incorporating one’s family, friends, healthcare providers, religious leaders, and God. These results carry significant implications as they support consideration for implementing a framework with which clinicians and researchers can approach the subject of EoL to allow for better understanding and improvement of the current state of EoL and EoL care in northern Tanzania.

## Supporting information

S1 File (PDF)

## Abbreviation

ACP: Advance Care Planning
EoL: End-of-Life
FGD(s): Focus Group Discussion(s)
HICs: High-Income Countries
HCP(s): Healthcare Professional(s)
KCMC: Kilimanjaro Christian Medical Centre
LMIC: Low and Middle-Income Country
QoD: Quality of Death
