# Supplementary material for: Good death: An exploratory study on perceptions and attitudes of patients, relatives, and healthcare providers, in northern Tanzania
Source: PLoS One. 2020 Jul 10;15(7):e0233494. doi: 10.1371/journal.pone.0233494 (PMC7351142; doi:10.1371/journal.pone.0233494)
Supplement: S1 File — (PDF) [file pone.0233494.s001.pdf]

APPENDIX 1. INFORMED CONSENT IN ENGLISH

Form
M0345

DUKE UNIVERSITY HEALTH SYSTEM

Kilimanjaro Christian Medical Centre

TUMAINI UNIVERSITY KILIMANJARO CHRISTIAN
MEDICAL COLLEGE

Consent to Participate in a Research Study:

Developing Capacity to Improve Care Transitions for Injury Patients in Tanzania: Focus Groups

INTRODUCTION

You are being asked to take part in this research study because either you are a patient, family member, friend or a healthcare provider of a patient who was treated at Kilimanjaro Christian Medical Centre. This study is being conducted by Dr. Blandina Mmbaga, Kilimanjaro Christian Research Institute, Dr. Mark Mvungi, Emergency Department, Kilimanjaro Christian Medical Center and Dr. Catherine Staton of the Division of Emergency Medicine at Duke University.

Research studies are voluntary and include only people who choose to take part. Please read this consent form carefully and take your time making your decision. As your study staff member discusses this consent form with you, please ask him/her to explain any words or information that you do not clearly understand. The nature of the study, risks, inconveniences, discomforts, and other important information about the study are listed below. You are free to ask questions about this study at any time. If you agree to take part in this study, you will be asked to sign and date this consent form. You will get a copy to keep.

WHY IS THIS STUDY BEING DONE? The purpose of this study is to understand disabilities and needs of patients who were treated at KCMC. The study will determine the functional, cognitive, psychological disabilities, pain and, if appropriate, end-of-life care needs or perceptions for patients who are seen at KCMC.

WHAT DO I HAVE TO DO IF I AM IN THIS STUDY?
If you agree to participate, we will discuss in a focus group setting the needs and disabilities suffered by patients after they are discharged from the hospital and, if appropriate, end-of-life care needs or perceptions for patients who are seen at KCMC. Our discussion will take about 60 minutes of your time.

HOW MANY PEOPLE WILL TAKE PART IN THIS STUDY?
About 100 people will participate in this study.

HOW LONG WILL I BE IN THIS STUDY?
Most likely your interview will last about 60 minutes.

WHY WOULD THE DOCTOR TAKE ME OFF THIS STUDY EARLY?
The study could be ended early by the Ministry of Health in Tanzania or by the Ethics Committee of KCMC. Ethics Committees and Institutional Review Boards watch over the safety and rights of research subjects. Also, the study could be ended early by the following groups in the United States, the Duke University Health System Institutional Review Board, and the Office of Human Research Protections.

WHAT ARE THE RISKS AND BENEFITS OF THE STUDY?
There is no benefit to you for participating in this study. There are no physical risks associated with this study. There is, however, the potential risk of loss of confidentiality. Every effort will be made to keep your, your family member/friend’s, or patient’s information confidential; however, this cannot be guaranteed. Some of the questions we will ask you as part of this study may make you feel

uncomfortable. You may refuse to answer any of the questions and you may take a break at any time during the study. You may stop your participation in this study at any time.

If your answers to questions show that you, your family member or friend, or your patient needs further medical or social assistance, we are obligated to refer him/her to an appropriate medical professional for treatment. We will not pay for the costs of this medical treatment or transport costs associated with these additional treatments.

CONFIDENTIALITY

Study records will be kept confidential as required by law. Your records will be assigned a unique study number. Information that links your name to the study number will be kept in a locked cabinet that can only be accessed by members of the research team. No personal identifiers will be sent to or used at Duke. If information from this study is presented at scientific meetings or in scientific journals, your identity or that of your family member/friend's, or your patient's will not be revealed.

WHAT ARE THE COSTS TO ME?

There is no additional cost to you for taking part in this research study.

WILL I RECEIVE ANY PAYMENTS?

There will be no financial compensation for participating in this study.

WHAT ABOUT RESEARCH RELATED INJURIES?

Immediate necessary care and support is available if an individual is injured because of participation in this research project, however, there is no provision for free medical care or for monetary compensation for such an injury. For questions about the study or research-related injury, contact Dr. Mark Mvungi from KCMC at (255) 78 62 40 988 or at (255) 75 43 01 149

VOLUNTARY PARTICIPATION/RIGHT TO WITHDRAW

You may choose not to be in the study, or, if you agree to be in the study, you may withdraw from the study at any time. If you agree to participate, you may refuse to answer any question or stop the interview at any time.

Your decision to not participate or to withdraw from the study will not involve any penalty or loss of benefits to which you are entitled, and will not affect your access to health care at KCMC, or that of your family member/friend's or your patient's. If you do decide to withdraw, we ask that you contact Dr. Blandina Mmbaga in writing and let her know that you are withdrawing from the study. Her mailing address is KCMC-Duke Collaboration, Box 3010, Sokoine Road, Moshi.

We will tell you about new information from this or other studies that may affect your health, welfare or willingness to stay in this study. If you want the results of the study, let the study staff know.

DUKE UNIVERSITY HEALTH SYSTEM

Kilimanjaro Christian Medical Centre

TUMAINI UNIVERSITY KILIMANJARO CHRISTIAN
MEDICAL COLLEGE

Consent to Participate in a Research Study:

Developing Capacity to Improve Care Transitions for Injury Patients in Tanzania: Focus Groups

WHAT DO I DO IF I HAVE QUESTIONS OR PROBLEMS?

For questions about the study or a research-related injury, or if you have complaints, concerns or suggestions about the research, contact Dr. Mark Mvungi from KCMC at (255) 78 62 40 988 or at (255) 75 43 01 149. For questions about your rights as a research participant, or to discuss problems, concerns or suggestions related to the research, or to obtain information or offer input about the research, contact the Kilimanjaro Christian Medical Centre (KCMC) Ethics Committee at telephone number (255) 27 27-53909 or the Duke University Health System Institutional Review Board at +1-919-668-5111.

STATEMENT OF CONSENT

"The purpose of this study, procedures to be followed, risks and benefits have been explained to me. I have been allowed to ask the questions I have, and my questions have been answered to my satisfaction. I have been told whom to contact if I have additional questions. I have read this consent form and agree to be in this study with the understanding that I may withdraw at any time. I have been told that I will be given a signed and dated copy of this consent form to keep."

Participant's Name (Print) Participant's Signature and Date

____

Study staff's Name Study staff's Signature and Date

____

Witness Name Witness Signature and Date

____

Appendix 2: Focus Group Prompts for Patients - English

INTRODUCTION

"We would like to welcome you to this Focus Group about perceptions of, and attitudes towards, planning for end of life care in Moshi.

None of us can predict death and it is ultimately up to God. We want to have a medical environment where we make sure that we are respecting the wishes of our patients, and their families, when it comes to their care near the end of life. To do this, we need to have a better understanding of how one defines a "good death" in Moshi. We also need to understand the general thoughts and feelings about the process of dying and planning for the end of life. We cannot understand these things unless we talk to community members, patients, families, friends, healthcare workers, religious leaders and many other groups. This is the reason we are having many focus groups, including this one. You all have many different experiences, we want to hear your own thoughts and experiences.

We will have a 60-90-minute discussion and we will audiotape you during this time period. We will then transcribe the discussion and translate it. You are welcome to participate and there will be no consequences should you decide to stop participating. There will be no monetary compensation for your time, but we will reimburse transportation costs and provide snacks and drinks at the conclusion of this discussion. Please ask me any questions about the process and the Focus Group today. If you have no further questions, please sign the informed consent sheet that we have distributed, and we will start soon."

I will tell you a story. Imagine you live in the community where it happened.

Story:

There is an elderly man, Bwana TJ Smith, who has had memory loss for many years. He and his wife, who died a few years ago, had 5 children and he lives with his only daughter, Stacy, and her family. Even though Bwana TJ has many medical problems and has been admitted to the hospital many times, he has been able to live a simple and good life. One morning, his condition suddenly worsens, and he is unable to speak. Stacy is worried that he might be dying soon, and she wants to call his priest because she feels this is what her father would have wanted. She feels he would like to die at peace with God and join his wife.

She calls her older brother, Bwana TJ's first child, to inform him that their father may be dying soon. The first child, TJ Junior, lives in Dar es Salaam and has not spoken with his father in 10 years after they had a big argument. Upon hearing about his father's condition, he tells his sister to take their father to take their father to KCMC immediately. He tells her not to worry about money because he can afford it. He regrets that he did not reconcile with his father earlier and can't bear the thought of him dying while their relationship is still damaged. Stacy is conflicted; she does not know what her father would have wanted.

The other children show up and, unfortunately, a quarrel starts. While this is going on, Bwana TJ Smith passes away.

1. What are your thoughts and feelings about this story? Please explain.
2. In Moshi, do people think there is such a thing as a good death or a bad death? If yes, what are the differences?
    a. Please give us examples of good or bad deaths.
    b. What is the role of spirituality or religion to a dying person and his/her family?
    c. How important, and why, is the place of death?
    d. How important is the funeral or burial ceremony?

Going back to the case of Bwana TJ.

3. What could have been planned in advance to prepare everybody for his dying?
    a. Would it have been appropriate for Bwana TJ's doctor to have offered him the opportunity to plan for his dying?
    b. Would it have been appropriate for Bwana TJ to have documented, in advance, his preferences and wishes for when he was near the end of life?

Changing topics, a little, I now want to ask you some questions about your illness.

4. You all have different ailments and we want to know, after you were informed about the diagnosis, what went through your mind?
    a. How important, and why, was it to ask about the future course of your disease, including treatment options, complications and anticipated needs?
    b. How important, and why, was it to let your physician know about your religious or spiritual beliefs?
    c. How important is it to express your, or your family's, priorities in life? And why?
        i. What about your, or your family's, concerns about what may happen in the future? And why?
        ii. What about your, or your family's, treatment preferences? And why?

5. As I said earlier, none of us can predict our death and our fate is in God's hands. But what if you suddenly found yourself nearing the end of life, what things would you consider important?

Appendix 3: Focus Group Prompts for Family, Friends or Social Actors - English

*[Introduction and Vignette are the same as in the Patient FG Guide]

Questions

1. What are your thoughts and feelings about this story? Please explain.
    a. Would it have been appropriate for Bwana TJ Smith to have documented, in advance, his preferences and wishes for when he was near the end of life?

2. In Moshi, do people think there is such a thing as a good death or a bad death? If yes, what are the differences?
    a. Please give us examples of good or bad deaths.
    b. Can you think of circumstances in which living longer might be more difficult than dying?
    c. What is the role of spirituality or religion to a dying person?
    d. What is the role of the family and friends when somebody is very sick or is about to die?

Changing topics, a little, I now want to ask you some questions about your loved one.

3. After you were informed about your loved one's diagnosis, what went through your mind?
    a. How important, and why, was it to ask about the future course of the disease, including treatment options, complications, and anticipated needs?
    b. What concerns do you have about the future?
    c. Without changing the diagnosis, what is your goal for your loved one's quality of life?
    d. What has been your role during the illness journey? Please give us examples of responsibilities you have had to bear so far.
    e. What kinds of conversations have you had with the patient or other family members?

4. As I said earlier, none of us can predict our death and our fate is in God's hands. But what if one of your family suddenly found his/herself nearing the end of life, what things would you consider important?

Appendix 4: Focus Group Prompts for Healthcare Workers- English

INTRODUCTION

“We would like to welcome you to this Focus Group about perceptions of, and attitudes towards, advance care planning in Moshi. This research study is being conducted by Dr. Catherine Staton and Dr. Blandina Mmbaga from the Kilimanjaro Christian Medical Center and Duke University Collaboration. The purpose of this research is to understand how one defines a “good death” in Moshi and the general perspectives on the process of dying and planning for the end of life. We thank you for participating. We will have a 60 - 90-minute audiotaped discussion on these topics. We will then transcribe the discussion and translate it. You can stop your participation at any time. There will be no monetary compensation for your time, but we will reimburse transportation costs and provide snacks and drinks at the conclusion of this discussion. There will be no consequences should you decide to stop participating in this discussion. All participants likely have had many different experiences, we want to hear your own thoughts and experiences.

Please ask me any questions about the process and the Focus Group today. If you have no further questions, please sign the informed consent sheet that we have distributed, and we will start in a few minutes."

1. From your experiences caring for patients, do you think there is such a thing like a good death or a bad death? If yes, what are the differences?
    a. Please give us examples of good or bad deaths.

2. Imagine you are taking care of a patient with a serious illness who has only one year of life left, what would your general approach to that patient’s care be?
    a. What do you think about talking with a patient, or with their close family members, about their diagnosis and the predicted course of the disease?
    b. What do you think is important to discuss with this patient, or the family? And why?
        i. What about priorities for the remainder of this patient’s life; concerns about what may happen in the future; desires for place of death; or treatment preferences?

3. Clinical Vignette:

You are looking after a Masaai herdswoman who has breast cancer and, 3 months earlier, a CT scan showed that the cancer had spread to the brain.

She has just been admitted and, this time, she came to the hospital with delirium, slurred speech, and yellow skin. She also has a headache, drowsiness and nausea. An abdominal ultrasound shows multiple liver abnormalities and the working diagnosis is progressive breast cancer. The cancer care team is evaluating her for potential palliative chemotherapy and supportive care in Moshi.

She has 15 children and her daughters, who she lives with, want her to be discharged this morning because they see that her condition is getting worse. They would like to take her to a church to have evil spirits removed; they believe these spirits are causing their mother’s symptoms.

a. What are your thoughts and feelings about this case?

The patient’s first child, who lives in Dar es Salaam and is a University graduate, contacts the hospital to say that he wants to bring his mother to Ocean Road Cancer Institute. He says this is what his mother would have wanted especially since he can afford it. He wants you to do “everything possible” to keep his mother alive until he can come to Moshi.

The other children cannot be contacted at this time. While debating how to proceed next, the patient begins choking and, unfortunately, passes away.

b. What could have been done for this patient?
c. What could have made the death ‘better’? Please explain your thoughts.
d. Would it have been appropriate to have offered the patient the opportunity to plan for this moment? What about these two options:
    - 3 months earlier, when it was suspected that the cancer had spread to the brain or, 1 year ago, when she was diagnosed with breath cancer?
e. Would it have been appropriate to have documented, in advance, her preferences and wishes regarding medical care at the end of her life?
f. The patient may have come across many healthcare workers during her illness journey – doctors, nurses, social workers, religious or spiritual figures, community health workers, and more. Which one of these people would have been the best person to initiate this type of discussion on planning for end of life care?
Why so?
g. How do you think the patient’s family will be affected by this loss? What kind of support would you consider offering or recommending to them?

Appendix 5. Research Team

Student Researcher:

Temitope Gafaar MD
Class of 2019,
Duke University School of Medicine
tog2@duke.edu
+1 (240) 416 6499

Research Mentors & Co-Investigators

Catherine A. Staton, MD MScGH
Assistant Professor of Surgery
Assistant Research Professor of Global Health
Director, Section of EM Global Health
Division of Emergency Medicine
Department of Surgery
Duke Global Health Institute
catherine.Staton@duke.edu
+1 (908) 872-9208

Oliver Henke, MD
Consultant and Lecturer,
Palliative Care and Department of Oncology,
Cancer Care Center,
Kilimanjaro Christian Medical Center
henke.Oliver@gmx.de

Msafiri Pesambili
Research Assistant & Data Analyst,
Casualty Research Team,
Kilimanjaro Christian Medical Center and Duke University Collaboration
msape05@gmail.com

Joao Ricardo Nickenig Vissoci, PhD
Assistant Professor in Surgery
Division of Emergency Medicine
Department of Surgery
Duke Global Health Institute
jnv4@duke.edu
+1 (919) 797 9228

Professor Blandina Theophil Mmbaga, MD, PhD
Director, Kilimanjaro Clinical Research Institute
Physician, Kilimanjaro Christian Medical Center
Lecturer, Kilimanjaro Christian Medical University College
b.mmbaga@kcri.ac.tz

Research Team: Nurses & Assistants

Sister Amina Mlay
Sister Elizabeth Ngowi
Joseph Kilasara

Appendix 5 (contd.)

Sister Anna Mchaki
Sister Evangeline Msanya
Julius Raymond Kingazi
Anthony Fredrick Monyo
Victoria Gerald Macha
Yvonne John Sawe
Sister Germana Lello

Participant Recruitment augmented by:
Sister Elizabeth Msoka
Sister Anna Massawe

Administrative and Accounting Support
Lysa Mackeen
Vera Wright
Cynthia Asiyo
Ashley Phillips
Frank Michael

Appendix 6
Recruitment for Patient Focus Groups

Inclusion Criteria: Participants must be ≥18 years of age and must have a life-threatening diagnosis, for example:

- HIV/AIDS,
- Malignancy/Cancer (all cases),
- Life-threatening Injury
- Advanced kidney disease,
- Advanced liver failure,
- Severe congestive heart failure (CHF),
- Advanced lung disease

Another way we can define a life-threatening diagnosis for this study is if:
The physician or healthcare provider thinks that this patient might die within one year

Additionally:

- Participants must be aware of the diagnosis and prognosis and they should be willing/able to talk about their experiences and perceptions
- they must be clinically sober at the time of enrollment and during the focus group;
- they must be medically stable;
- they must be able to communicate in Swahili or English;
- and they must consent to participate.

Exclusion Criteria: Participants will be excluded from the focus groups if they are medically unstable or have an acutely deteriorating condition, are too critically ill to participate, do not speak English or Swahili, are <18 years of age, or do not consent to be enrolled.
